# Reconstructing the Developmental Trajectories of Multiple Subtypes in Pulmonary Parenchymal Epithelial Cells by Single-Cell RNA-seq

**DOI:** 10.3389/fgene.2020.573429

**Published:** 2020-10-06

**Authors:** Yiwei Huang, Yuansheng Zheng, Jiacheng Yin, Tao Lu, Ming Li, Jiaqi Liang, Zhengyang Hu, Guoshu Bi, Cheng Zhan, Liang Xue, Wei Jiang, Qun Wang

**Affiliations:** Department of Thoracic Surgery, Zhongshan Hospital, Fudan University, Shanghai, China

**Keywords:** lung tissue, epithelial cells, developmental trajectories, single-cell RNA-seq, stem cell

## Abstract

**Background:**

Some lung diseases are cell type-specific. It is essential to study the cellular anatomy of the normal human lung for exploring the cellular origin of lung disease and the cell development trajectory.

**Methods:**

We used the Seurat R package for data quality control. The principal component analysis (PCA) was used for linear dimensionality reduction. UMAP and tSNE were used for dimensionality reduction. Muonocle2 was used to extract lung epithelial cells to analyze the subtypes of epithelial cells further and to study the development of these cell subtypes.

**Results:**

We showed a total of 20154 high quality of cells from human normal lung tissue. They were initially divided into 17 clusters cells and then identified as seven types of cells, namely macrophages, monocytes, CD8 + T cells, epithelial cells, endothelial cells, adipocytes, and NK cells. 4240 epithelial cells were extracted for further analysis and they were divided into seven clusters. The seven cell clusters include alveolar cell, alveolar endothelial progenitor, ciliated cell, secretory cell, ionocyte cell, and a group of cells that are not clear at present. We show the development track of these subtypes of epithelial cells, in which we speculate that alveolar epithelial progenitor (AEP) is a kind of progenitor cells that can develop into alveolar cells, and find six essential genes that determine the cell fate, including AGER, RPL10, RPL9, RPS18, RPS27, and SFTPB.

**Conclusion:**

We provide a transcription map of human lung cells, especially the in-depth study on the development of epithelial cell subtypes, which will help us to study lung cell biology and lung diseases.

## Introduction

In mammals, the lung is a highly branched network, in which the distal area of the bronchus tree transforms into dense alveolus vesicles during development. The lung plays a crucial role in both gas exchange and mucosal immunity. Its anatomical structure provides these functions through the following ways: (1) guiding air into the airway of the respiratory unit, providing mucociliary clearance, and forming a barrier to inhaled particles and pathogens; (2) alveoli, the distal cystic structure where gas exchange takes place (Vieira Braga et al., 2019). The lung is a complex organ with long development time. It is controlled and coordinated by gene network and dynamic crosstalk of multiple cells, including lineage assignment, cell proliferation, differentiation, migration, morphogenesis, and damage repair. The function of epithelial tissue depends on the abundance and distribution of differentiated cells (Rock and Hogan, 2011; Hines and Sun, 2014). Researchers have been trying to restore tissue function after lung injury, which requires understanding how physiological tasks are distributed among cell types and how cell states change between homeostasis, damage/repair, and disease (Rock et al., 2010).

Lung diseases, including lung trauma, viral pneumonia, lung cancer, asthma, chronic obstructive pulmonary disease, and cystic fibrosis, need stem cells to restore the damaged lung tissue after a large number of lung tissue cells lose function (Monsel et al., 2016). Finding stem cells of lung tissue is of great significance for the treatment of lung diseases. In this study, we integrated and analyzed samples of single-cell RNA sequencing (scRNA-seq) from multiple normal lung tissues to explore progenitor cells that may regenerate into alveolar epithelial cells. The study of normal lung tissue cells will help us to better understand the process of lung development, especially the process of alveolar formation. It will have a significant impact on the survival rate of premature infants with incomplete lung development, and will also promote the repair and regeneration of lung injury in adults.

## Results

**FIGURE 1:**
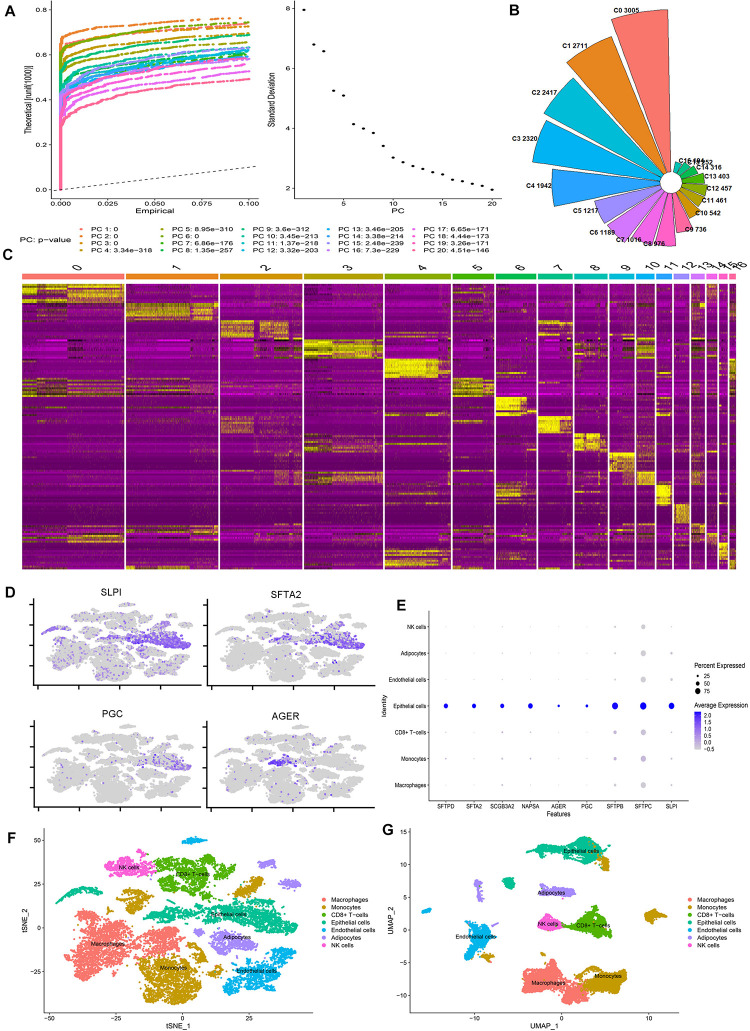
scRNA-seq reveals the cell populations of the human lung. **(A)** Jackstraw plot and Elbow plot show the *p*-value distribution of each principal component (PC). **(B)** Nightingale’s rose diagram showed the proportion of each lung cell type. **(C)** Heatmap showing the differentially expressed gene of each cluster. **(D)** Scatter plot of classic marker genes in epithelial cells. **(E)** Bubble plots of the first nine marker genes identified in our study. **(F,G)** tSNE and UMAP plots showing all the identified cell types distribution.

We calculated the number of genes in each cell, the number of UMIs (Unique Molecular Identifiers), and the percentage of mitochondrial genes (Supplementary Figure 1A), and the correlation plot of sequencing depth (Supplementary Figure 1C). Using the FindVariableFeatures function, we extracted 2000 genes with a large coefficient of variation between cells (Supplementary Figure 1B). After QC, we furtherly analyzed a total of 20154 high-quality lung cells from four normal lung tissue. To eliminate the interference of cell cycle on cell dimensionality reduction clustering, we analyzed the influence of cell cycle on clustering and removed cell cycle-related genes. The final data results show that the cells of each cell cycle are evenly distributed in each cell cluster (Supplementary Figures 1E,F). The results of the principal component analysis (PCA) show that the first 20 pcs have good discrimination (Figure 1A). All of them are included in the next step of the analysis. We also show the gene expression heatmap of the first four PCs and the related genes of each PCA component (Supplementary Figures 3A,B).

After removing the batch effect by harmony, the cells from each sample are evenly distributed in each cell cluster (Supplementary Figures 3C,D). We identified 17 clusters of 194–3005 cells per cluster (Figure 1B). Based on the expression of the top ten genes with the most significant differential expression in each cell cluster, we drew the heatmap of marker gene expression in these 17 cell clusters (Figure 1C). Cell clusters were visualized using two different methods (UMAP and tSNE), and the results were the same (Supplementary Figures 3E,F). According to the specific marker gene (Supplementary Table 2), we subdivide the cells into seven categories: macrophages, monocytes, CD8 + T cells, epithelial cells, endothelial cells, adipocytes, and NK cells (Figures 1F,G). Our scatter plot, bubble plot, and violin plot of the marker genes of epithelial cells show that these genes can distinguish epithelial cells from other cells well (Figures 1D,E and Supplementary Figures 3G,H). We also found that the classic maker genes of almost all epithelial cells are highly expressed in our epithelial cells (Supplementary Figure 4E), so we think these results are reliable.

**FIGURE 2:**
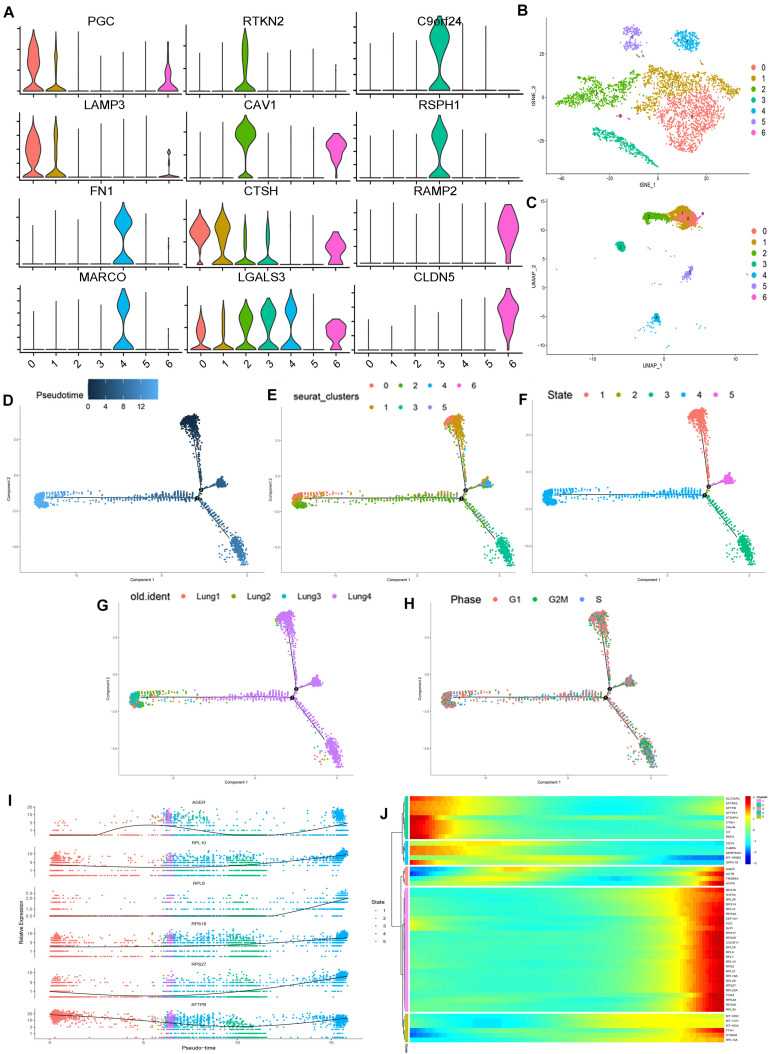
Subpopulations of epithelial cells and reconstructing the developmental trajectory of epithelial cells. **(A)** Violin plots showing the expression of marker genes in each cluster of epithelial cells. **(B,C)** tSNE and UMAP plots showing all the epithelial cell subtypes distribution. **(D)** Pseudotemporal trajectory of seven subtype cells. **(E)** The gradual change from black to light blue indicates the change of pseudotime. **(F)** The pseudotime trajectory showed epithelial cells with five different states. **(G)** The trajectory showing the subtype cells come from four lung samples. **(H)** The trajectory showing the phase distribution of all cells in different branches. **(I)** According to the changes of gene expression in pseudotime, the top six genes that may affect the fate of cells were shown. **(J)** Heatmap showed the top 50 genes that affect cell fate decisions. The 50 genes were divided into five clusters.

Then, we extracted the lung epithelial cells for further analysis. Our results show that there are 4240 epithelial cells. After dimensionality reduction clustering of these cells, we found that epithelial cells can be divided into seven different clusters (Figures 2B,C). Violin plots showing the expression of marker genes in each cluster of epithelial cells (Figure 2A). We found out the maker genes of each cluster. Then, we drew the heatmap of the top ten differentially expressed marker genes (Supplementary Figure 4D), among which the representative marker genes in each cluster were displayed with a violin plot and bubble plot (Supplementary Figures 4F,G). By referring to the literature and CellMarker website, we have identified several types of epithelial cells, including alveolar cell (SFTPC+, NAPSA+, SFTPD+, PGC+, SFTPA1+), alveolar epithelial progenitor (AEP) (TM4SF1+, SLC34A2−, ABCA3−, CAV1+, AGER+, RTKN2+) (Zacharias et al., 2018), ciliated cell (C20orf85+, C11orf88+, C1orf192+, ATPIF1+, ALDH3B1+), secretory cell (SCGB1A1+, HLA-DRA+, CD74+, HLA-DRB1+, CTSC+), ionocyte cell (FXYD6+, TPM1+, ID3+, JADE1+, TMEM160+), and a class of cells (SFTPC−, NAPSA−, SFTPD−, PGC−, SFTPA1−) which are not clear at present (Montoro et al., 2018; Plasschaert et al., 2018; Zacharias et al., 2018; Vieira Braga et al., 2019; Table 1 and Supplementary Table 2). Besides, we used Monocle2 to perform the pseudo time trajectories of all epithelial cells and showed the fate determination between them (Figures 2D–H). The shade of the color indicates the trajectory of cell development, which is sorted according to the pseudo-time value (Figure 2D). We mapped the Seurat cluster ID of epithelial cells to the distribution of the pseudo-time value of each cell to show the development trajectory between different subgroups of epithelial cells (Figure 2E). Five branches with different colors were obtained by pseudo-time analysis, which represented the fate of different cell subsets in epithelial cells (Figure 2F). We mapped epithelial cells from different tissue sources and the cell cycle of these cells to the pseudo temporal trajectory to determine the influence of tissue source (Figure 2G) and cell cycle on the pseudo-time analysis (Figure 2H). In combination with the results of the pseudo-time analysis and the above annotation of epithelial cell subsets, we speculate that AEP, as a stem cell, might have the potential to differentiate in multiple directions. We found two subtypes (0 and 1 cell clusters) in alveolar cells. Interestingly, there was a kind of cell cluster in pulmonary epithelial cells, which is not clear yet, according to the existing literature. In this unclear cell cluster, the expression of marker genes was characterized by the negative expression of some specific maker genes as the positive expression in the alveolar cells. At the same time, we found the first six genes that affect cell fate (Figure 2I), including AGER, RPL10, RPL9, RPS18, RPS27, and SFTPB. To show more detailed genetic information, we show the top 50 genes that affect the fate of cells (Figure 2J). These genes may play an important role in the direction of cell differentiation.

**TABLE 1:** Annotation of pulmonary epithelial subtypes cells based on the maker gene.

**Cluster**	**Gene name**	**Avg_logFC**	***P*_val_adj**	**Cell type**
0&1	SFTPC	1.193320	0	Alveolar cell
0&1	NAPSA	0.983520	3.59E−281	Alveolar cell
0&1	SFTPD	0.948704	7.31E−262	Alveolar cell
0&1	PGC	1.706521	3.94E−257	Alveolar cell
0&1	SFTPA1	0.954615	1.78E−256	Alveolar cell
2	TM4SF1	0.528425	4.67E−16	Alveolar epithelial progenitor
2	SLC34A2	–0.748240	1.82E−39	Alveolar epithelial progenitor
2	ABCA3	–1.095113	3.20E−48	Alveolar epithelial progenitor
2	CAV1	2.503099	0	Alveolar epithelial progenitor
2	AGER	3.030825	2.40E−300	Alveolar epithelial progenitor
2	RTKN2	2.606841	1.01E−269	Alveolar epithelial progenitor
3	C20orf85	2.580915	0	Ciliated cell
3	C11orf88	2.550691	0	Ciliated cell
3	C1orf192	1.719045	8.41E−210	Ciliated cell
3	ATPIF1	1.266959	1.16E−32	Ciliated cell
3	ALDH3B1	0.915747	4.78E−16	Ciliated cell
4	SCGB1A1	0.901010	1.74E−130	Secretory cell
4	HLA-DRA	1.664870	9.27E−95	Secretory cell
4	CD74	1.131092	1.15E−52	Secretory cell
4	HLA-DRB1	1.312359	7.95E−36	Secretory cell
4	CTSC	1.556552	1.18E−11	Secretory cell
5	SFTPC	–1.587732	2.03E−19	Unknow
5	NAPSA	–1.136357	1.45E−31	Unknow
5	SFTPD	–1.272810	2.83E−33	Unknow
5	PGC	–1.979828	8.22E−21	Unknow
5	SFTPA1	–0.794656	4.12E−09	Unknow
6	FXYD6	0.637504	3.26E−81	Ionocyte cell
6	TPM1	1.092610	6.05E−29	Ionocyte cell
6	ID3	1.361644	1.88E−20	Ionocyte cell
6	JADE1	0.656350	2.71E−18	Ionocyte cell
6	TMEM160	0.710465	4.95E−16	Ionocyte cell

## Discussion

In recent years, with the application of scRNA-seq (Treutlein et al., 2014; Ardini-Poleske et al., 2017; Nabhan et al., 2018) technology in lung tissue, it has been widely used to describe the cell type diversity and lineage level in the lung tissue due to its advantages in obtaining single-cell information. These studies have contributed to find new cell subtypes and explain the development track between these cells in adult lung tissue, so as to find out the stem cells (Zacharias et al., 2018). At present, the most studied lung stem cells are the basal cells of the tracheal epithelium. The basal progenitor cells are thought to be able to regenerate into a variety of epithelial components. It is necessary for restoring normal organ homeostasis by regeneration of functional tissue after severe trauma (Zacharias et al., 2018). One of the most important parts of lung regeneration is alveoli. In this study, we found that alveolar epithelial cells have two types of cells (0 and 1 cell clusters in epithelial cell subtypes), which is consistent with previous studies. We think that this may be due to some differences in expression between the two subtypes of alveolar cells. There are two types of alveolar epithelial cells, type 1 and type 2. The expression of their maker genes such as SFTPC, NAPSA, SFTPD, PGC, and SFTPA1 is consistent between these two subgroups. This is also consistent with the results of the heatmap of maker genes in cluster 0 and cluster 1 in Supplementary Figure 3.

Previous studies showed that there are significant differences in cell components between airway and lung parenchyma, and also in different parts of the airway (Rock et al., 2009). The basal cells distribute in the trachea and bronchi. The secretory cells distribute in the bronchi and bronchioles (Rock et al., 2009). The alveolar stem cells distribute in the junction of the bronchioloalveolar tubes (Lee et al., 2014). The type II alveolar epithelial cells distribute in the alveolar area (Barkauskas et al., 2013). Lung injury activates specialized adult epithelial progenitor cells to regenerate the epithelium. According to the degree of injury, the remaining alveolar type II cells and distal airway stem/progenitor cells will mobilize to cover the stripped alveoli and restore the normal barrier. In addition to basal cells, the main source of airway stem/progenitor cells is still uncertain. Researchers found a unique subpopulation (about 5%) of rod-shaped lineage negative epithelial progenitor cells characterized by the high expression of H2-K1, which is essential for alveolar repair. The resting H2-K1 cells are the source of regeneration activity of almost all airway lineages *in vitro*. Normal H2-K1 (high) cells can differentiate into alveolar cells and recover lung function after transplantation into the damaged lung. These findings suggest that small subpopulations of specialized stem/progenitor cells are necessary for effective lung regeneration and are potential therapeutic adjuncts after major lung injury (Kathiriya et al., 2020). Meanwhile, researchers from the Perelman School of Medicine at the University of Pennsylvania identified a lung progenitor cell that repairs the alveoli. They isolated these progenitors from the lungs of mice and humans and described their characteristics (Zacharias et al., 2018). Morrisey’s team identified an AEP, in which AEP was found in a larger population of cells called alveolar type 2 cells (Zacharias et al., 2018). In this study, according to the maker gene of AEP cells reported in the literature, we have also identified such cells in normal lung epithelial cells, including TM4SF1+, SLC34A2−, ABCA3−, CAV1+, AGER+, RTKN2+, etc. Besides, we can see that 0/1/2 of these cell clusters are very similar in the map of UMAP clustering, which is also consistent with the discussion that AEP may be mixed in alveolar epithelial cells as reported above.

The transition area between the smallest terminal bronchioles and alveoli is called the junction of bronchioloalveolar ducts. This part of the mouse body contains ciliated cells and secretory cells, but the cells distributed in this part of the human body are not clear (Cuzić et al., 2012). A small number of cells (1–2 cells in each trachea) at the junction of the bronchioloalveolar duct were labeled as SCGB1A1 (secretory cells) and alveolar surfactant protein C (SFTPC), which is a unique protein of type II alveolar epithelial cells (Rawlins et al., 2009). The conclusion is that these double-positive cells are bronchioloalveolar stem cells. In our study, we did not find the SCGB1A1 and SFTPC double-positive cells, but we identified the cell cluster 2 of AEP cells, whose SFTPC expression was high. We speculate that the possible reason for this is that the number of such stem cells is relatively small, and there are many experimental processes and data analysis processes maybe will lead to the loss of some cells. For example, some cells will be filtered out in the process of making single cell suspension and single cell data quality control. In this way, the relatively small number of such stem cells may not be recognized effectively. Maybe we can find these cells in a larger sample size, which needs further study in the future.

In recent years, the research of stem cell and progenitor cell lineage recognition and the activity of these cells in adults has developed rapidly (Rath et al., 2018). In contrast, the research on the regulation of cell behavior by molecular level signaling pathway is slow. The major molecular signaling pathways used in the process of individual development play an important role in the process of lung cell repair (Hogan et al., 2014). However, these viewpoints need to be further tested in physiological or clinical related experimental models. To explore the mechanism of lung formation, especially in the field of alveolar formation, is in the ascendant. The gas-liquid interface culture system of bronchial epithelium and the *in vitro* organ-like culture system (bronchus and alveolus), which are popular in recent years, provide the best technical support and research platform for solving these problems in the field of respiratory stem/progenitor cells.

To sum up, we provide a transcription map of human lung cells, especially the in-depth study on the development of epithelial cell subtypes, which will help us to study the lung cell biology and the relationship between cell types and diseases.

## Materials and Methods

### Data Acquisition and Ethical Review

We downloaded GSE130148 and GSE132771 10x genomics RNA-seq datasets from the GEO database^1^, extracted the data of single-cell sequencing of normal lung tissue, and combined the two datasets with MergeSeurat function in Seurat (Satija et al., 2015; Stuart et al., 2019) R package (version 3.1.4)^2^ as our analysis data. The Institutional Review Committee of Zhongshan Hospital (Fudan University, Shanghai, China) approved this study to be exempted from research.

### Using Seurat for Data Quality Control (QC)

We use R (version 3.6.3)^3^ and Seurat R package for data quality control. Single-cell data sets may contain “uninteresting” sources of variation, including not only technical noise, but also batch effects, and even sources of biological variation (such as cell cycle stage) (Nagano et al., 2017). Regression of these signals from the analysis can improve the downstream dimension reduction and clustering (Buettner et al., 2015). Based on previous studies, the screening criteria of cells were determined (Zacharias et al., 2018; Vieira Braga et al., 2019). According to the median of genes and percentage of mitochondrial genes in lung samples (Supplementary Figure 1A), cells with <200 and >3000 genes (potential cell diploid) and percentage of mitochondrial genes >20% were screened and removed. After QC, we obtained a total of 20154 high-quality lung cells. The relationship between mitochondrial gene percentage and mRNA reading, and between mRNA quantity and mRNA reads were detected and visualized (Supplementary Figure 1C). After normalization of data, all highly variable genes in the single-cell data were identified after controlling the relationship between mean expression and dispersion (Supplementary Figure 1B). All variable genes (*n* = 18458) were used for downstream analysis, i.e., PCA.

### Analyze the Effect of Cell Cycle on Cell Clustering and Remove Cell Cycle-Related Genes

We used a set of 43 G1/S and 54 G2/M cell cycle-specific genes with known stage specificity to monitor, analyze, and quantify the cell cycle stages of each cell (Tirosh et al., 2016). The heterogeneity of cell cycle, especially the transition of mitotic cells between S-phase and G2/M-phase, drives a large number of transcriptome variations, thus concealing biological signals. We classified the cells according to the maximum average expression (“cycle fraction”) of the two groups of cell cycle genes. When the G1/S and G2/M Cycle scores are less than 2, we consider these cells as non-proliferative cells. Otherwise, we think the cells are proliferative. After cell cycle analysis, we observed no deviation caused by cell cycle genes (Supplementary Figures 1E,F).

### Principal Component Analysis

The PCA is a multivariate statistical method to investigate the correlation between multiple variables. It studies how to reveal the internal structure of multiple variables through a few principal components, that is, to derive a few principal components from the original variables, so that they retain as much information as possible about the original variables, and are not related to each other (Li et al., 2017). Generally, the mathematical treatment is to make the original indicators into a linear combination as a new comprehensive indicator. We use PCA with variable genes as input and recognize significant principal component (PC) based on jackstraw function. The first 20 pcs meet our requirements (Figure 2A), and all of them are selected for the next analysis.

### Data Secondary Analysis After Reducing the Batch Effect

Since this data comes from the results of two previous studies, which are four different batches of lung tissue samples, to avoid the impact of the batch effect on the downstream analysis, we adopted a strategy to alleviate the batch effect, called “harmony” (Tran et al., 2020). R package harmony focuses on scalable integration of scRNA-seq data for batch correction and meta-analysis (Tran et al., 2020) (version 1.0)^4^. We examined the batch effect among four different lung samples (Supplementary Figures 1D, 2C). At the resolution of 0.25, we use FindClusters function to cluster cells and divide them into 17 different cell types. Next, we use the FindAllMarkers function to find the differentially expressed genes between each type of cell (Supplementary Table 1).

### Cell Type Markers

First, we use the SingleR package (version 1.1.14)^5^ for cell type automatic annotation. Then, according to a widely used cell maker query website: CellMarker (Zhang et al., 2019)^6^, query the maker of the corresponding cell line, and query the maker in the previous research results to proofread (Montoro et al., 2018; Plasschaert et al., 2018; Zacharias et al., 2018; Vieira Braga et al., 2019). Finally, we annotated seven major lung tissue cells (Figures 2F,G).

### Reconstruction of EPI Cell Differentiation Track With Monocle2

We analyzed epithelial cell fate determination and pseudo time trace by monocle2 R software package (Qiu et al., 2017) (version 2.12.0)^7^. Based on the expression matrix of the single-cell transcriptome, monocle2 can simulate the biological process of a cell population by unsupervised learning. We import the data containing 4240 epithelial cells into Monocle2. We used genes expressed in at least ten cells and more than 5% of the cells. Then, we use the threshold of cell nearest distance (delta) and local density (Rho) to determine the number of clusters (Supplementary Figures 4B,C). Then, we analyzed the differential gene expression. We used the first 1000 genes with the most significant differential expression as the sequencing gene set and carried out the dimension reduction trajectory analysis.

## Data Availability Statement

The datasets presented in this study can be found in online repositories. The names of the repository/repositories and accession number(s) can be found in the article/Supplementary Material.

## Author Contributions

CZ, YH, and YZ: methodology. JY, TL, and ML: data acquisition. YH, JY, and JL: data analysis. YH, ZH, and GB: manuscript preparation. CZ, LX, QW, and WJ: management and direction. All authors contributed to the article and approved the submitted version.

## Conflict of Interest

The authors declare that the research was conducted in the absence of any commercial or financial relationships that could be construed as a potential conflict of interest.

## Footnotes

1 https://www.ncbi.nlm.nih.gov/gds/

2 https://satijalab.org/seurat/

3 https://www.r-project.org/

4 https://github.com/immunologics/harmony

5 http://www.bioconductor.org/packages/release/bioc/html/single.html/

6 http://biocc.hrbmu.edu.cn/cellmarker/index.jsp/

7 http://cole-trapnell-lab.github.io/monocle-release/

## Supplementary Material

Supplementary Figure 1 Quality control (QC) of human lung single-cell data. **(A)** Scatter plot illustrating the number of genes, unique molecular identifiers (UMIs), the percentage of mitochondrial genes in each cell, and the percentage of erythrocytic genes in each cell of four lung samples. **(B)** 2000 genes with a high coefficient of variation between cells (Red points). **(C)** The relationship between the percentage of mitochondrial genes and the mRNA reads, together with the relationship between the amount of mRNA and the reads of mRNA. **(D)** The batch effect between four different lung samples. **(E,F)** tSNE and UMAP plots showing the cell cycle status of all cells.

Supplementary Figure 2 Human lung single-cell data characteristics. **(A)** Before data quality control, scatter plot illustrating the number of genes, unique molecular identifiers (UMIs), the percentage of mitochondrial genes in each cell, and the percentage of erythrocytic genes in each cell of four lung samples. **(B)** Before data quality control, the relationship between the percentage of mitochondrial genes and the mRNA reads, together with the relationship between the amount of mRNA and the reads of mRNA. **(C)** After quality control, UMAP plots showing the batch effect between four different lung samples.

Supplementary Figure 3 Reduced dimension cluster analysis of scRNA-seq data of the human lung tissue. **(A)** Heatmap displayed the first four principal components (PCs). **(B)** The high variant genes in the first four PCs. **(C,D)** tSNE and UMAP plots showing the sample sources of cells between different cell clusters. **(E,F)** tSNE and UMAP plots showing lung tissue cells can be divided into 17 cell clusters. **(G)** Violin plots showing the expression of marker genes in epithelial cells. **(H)** Scatter plot of marker genes in epithelial cells.

Supplementary Figure 4 The expression features of subpopulations of human lung epithelial cells. **(A)** Feature genes were selected according to the average expression level of genes (≥0.1). **(B)** Variation curve of difference between each principal component (PC). **(C)** Results of the Density Peak Cluster clustering algorithm. **(D)** Heatmap showing the marker genes of each cluster of epithelial cells. **(E)** Scatter plot of classic marker genes in epithelial cells. **(F–J)** Bubble plots of the first nine marker genes in each cluster of epithelial cells, **(F)** cluster 0&1; **(G)** cluster 3; **(H)** cluster 4; **(I)** cluster 5; **(J)** cluster 6.

## References

[B1] Ardini-Poleske M. E. Clark R. F. Ansong C. Carson J. P. Corley R. A. Deutsch G. H. (2017). LungMAP: the molecular atlas of lung development program. *Am. J. Physiol. Lung Cell Mol. Physiol.* 313 L733–L740. 28798251 10.1152/ajplung.00139.2017 PMC5792185

[B2] Barkauskas C. E. Cronce M. J. Rackley C. R. Bowie E. J. Keene D. R. Stripp B. R. (2013). Type 2 alveolar cells are stem cells in adult lung. *J. Clin. Invest.* 123 3025–3036. 23921127 10.1172/JCI68782 PMC3696553

[B3] Buettner F. Natarajan K. N. Casale F. P. Proserpio V. Scialdone A. Theis F. J. (2015). Computational analysis of cell-to-cell heterogeneity in single-cell RNA-sequencing data reveals hidden subpopulations of cells. *Nat. Biotechnol.* 33 155–160. 10.1038/nbt.3102 25599176

[B4] Cuzić S. Bosnar M. Kramarić M. D. Ferencić Z. Marković D. Glojnarić I. (2012). Claudin-3 and Clara cell 10 kDa protein as early signals of cigarette smoke-induced epithelial injury along alveolar ducts. *Toxicol. Pathol.* 40 1169–1187. 10.1177/0192623312448937 22659244

[B5] Hines E. A. Sun X. (2014). Tissue crosstalk in lung development. *J. Cell. Biochem.* 115 1469–1477. 10.1002/jcb.24811 24644090 PMC8631609

[B6] Hogan B. L. Barkauskas C. E. Chapman H. A. Epstein J. A. Jain R. Hsia C. C. (2014). Repair and regeneration of the respiratory system: complexity, plasticity, and mechanisms of lung stem cell function. *Cell Stem Cell* 15 123–138. 10.1016/j.stem.2014.07.012 25105578 PMC4212493

[B7] Kathiriya J. J. Brumwell A. N. Jackson J. R. Tang X. Chapman H. A. (2020). Distinct airway epithelial stem cells hide among club cells but mobilize to promote alveolar regeneration. *Cell Stem Cell* 26 346–358. 10.1016/j.stem.2019.12.014 31978363 PMC7233183

[B8] Lee J. H. Bhang D. H. Beede A. Huang T. L. Stripp B. R. Bloch K. D. (2014). Lung stem cell differentiation in mice directed by endothelial cells via a BMP4-NFATc1-thrombospondin-1 axis. *Cell* 156 440–455. 10.1016/j.cell.2013.12.039 24485453 PMC3951122

[B9] Li H. Courtois E. T. Sengupta D. Tan Y. Chen K. H. Goh J. (2017). Reference component analysis of single-cell transcriptomes elucidates cellular heterogeneity in human colorectal tumors. *Nat. Genet.* 49 708–718. 10.1038/ng.3818 28319088

[B10] Monsel A. Zhu Y. G. Gudapati V. Lim H. Lee J. W. (2016). Mesenchymal stem cell derived secretome and extracellular vesicles for acute lung injury and other inflammatory lung diseases. *Expert Opin. Biol. Ther.* 16 859–871. 10.1517/14712598.2016.1170804 27011289 PMC5280876

[B11] Montoro D. T. Haber A. L. Biton M. Vinarsky V. Lin B. Birket S. E. (2018). A revised airway epithelial hierarchy includes CFTR-expressing ionocytes. *Nature* 560 319–324. 10.1038/s41586-018-0393-7 30069044 PMC6295155

[B12] Nabhan A. N. Brownfield D. G. Harbury P. B. Krasnow M. A. Desai T. J. (2018). Single-cell Wnt signaling niches maintain stemness of alveolar type 2 cells. *Science* 359 1118–1123. 10.1126/science.aam6603 29420258 PMC5997265

[B13] Nagano T. Lubling Y. Várnai C. Dudley C. Leung W. Baran Y. (2017). Cell-cycle dynamics of chromosomal organization at single-cell resolution. *Nature* 547 61–67. 10.1038/nature23001 28682332 PMC5567812

[B14] Plasschaert L. W. Žilionis R. Choo-Wing R. Savova V. Knehr J. Roma G. (2018). A single-cell atlas of the airway epithelium reveals the CFTR-rich pulmonary ionocyte. *Nature* 560 377–381. 10.1038/s41586-018-0394-6 30069046 PMC6108322

[B15] Qiu X. Hill A. Packer J. Lin D. Ma Y. A. Trapnell C. (2017). Single-cell mRNA quantification and differential analysis with Census. *Nat. Methods* 14 309–315. 10.1038/nmeth.4150 28114287 PMC5330805

[B16] Rath E. Moschetta A. Haller D. (2018). Mitochondrial function - gatekeeper of intestinal epithelial cell homeostasis. *Nat. Rev. Gastroenterol. Hepatol.* 15 497–516. 10.1038/s41575-018-0021-x 29844587

[B17] Rawlins E. L. Okubo T. Xue Y. Brass D. M. Auten R. L. Hasegawa H. (2009). The role of Scgb1a1+ Clara cells in the long-term maintenance and repair of lung airway, but not alveolar, epithelium. *Cell Stem Cell* 4 525–534. 10.1016/j.stem.2009.04.002 19497281 PMC2730729

[B18] Rock J. R. Hogan B. L. (2011). Epithelial progenitor cells in lung development, maintenance, repair, and disease. *Annu. Rev. Cell Dev. Biol.* 27 493–512. 10.1146/annurev-cellbio-100109-104040 21639799

[B19] Rock J. R. Onaitis M. W. Rawlins E. L. Lu Y. Clark C. P. Xue Y. (2009). Basal cells as stem cells of the mouse trachea and human airway epithelium. *Proc. Natl. Acad. Sci. U.S.A.* 106 12771–12775. 10.1073/pnas.0906850106 19625615 PMC2714281

[B20] Rock J. R. Randell S. H. Hogan B. L. (2010). Airway basal stem cells: a perspective on their roles in epithelial homeostasis and remodeling. *Dis. Model. Mech.* 3 545–556. 10.1242/dmm.006031 20699479 PMC2931533

[B21] Satija R. Farrell J. A. Gennert D. Schier A. F. Regev A. (2015). Spatial reconstruction of single-cell gene expression data. *Nat. Biotechnol.* 33 495–502. 10.1038/nbt.3192 25867923 PMC4430369

[B22] Stuart T. Butler A. Hoffman P. Hafemeister C. Papalexi E. Mauck W. R. (2019). Comprehensive integration of single-cell data. *Cell* 177 1888–1902. 10.1016/j.cell.2019.05.031 31178118 PMC6687398

[B23] Tirosh I. Izar B. Prakadan S. M. Wadsworth M. N. Treacy D. Trombetta J. J. (2016). Dissecting the multicellular ecosystem of metastatic melanoma by single-cell RNA-seq. *Science* 352 189–196. 27124452 10.1126/science.aad0501 PMC4944528

[B24] Tran H. Ang K. S. Chevrier M. Zhang X. Lee N. Goh M. (2020). A benchmark of batch-effect correction methods for single-cell RNA sequencing data. *Genome Biol.* 21:12. 10.1186/s13059-019-1850-9 PMC6964114 31948481

[B25] Treutlein B. Brownfield D. G. Wu A. R. Neff N. F. Mantalas G. L. Espinoza F. H. (2014). Reconstructing lineage hierarchies of the distal lung epithelium using single-cell RNA-seq. *Nature* 509 371–375. 10.1038/nature13173 24739965 PMC4145853

[B26] Vieira Braga F. A. Kar G. Berg M. Carpaij O. A. Polanski K. Simon L. M. (2019). A cellular census of human lungs identifies novel cell states in health and in asthma. *Nat. Med.* 25 1153–1163. 31209336 10.1038/s41591-019-0468-5

[B27] Zacharias W. J. Frank D. B. Zepp J. A. Morley M. P. Alkhaleel F. A. Kong J. (2018). Regeneration of the lung alveolus by an evolutionarily conserved epithelial progenitor. *Nature* 555 251–255. 10.1038/nature25786 29489752 PMC6020060

[B28] Zhang X. Lan Y. Xu J. Quan F. Zhao E. Deng C. (2019). CellMarker: a manually curated resource of cell markers in human and mouse. *Nucleic Acids Res.* 47 D721–D728. 30289549 10.1093/nar/gky900 PMC6323899

