# Supplementary figures and images for: Reconstructing the Developmental Trajectories of Multiple Subtypes in Pulmonary Parenchymal Epithelial Cells by Single-Cell RNA-seq
Source: Front Genet. 2020 Oct 6;11:573429. doi: 10.3389/fgene.2020.573429 (PMC7573224; doi:10.3389/fgene.2020.573429)

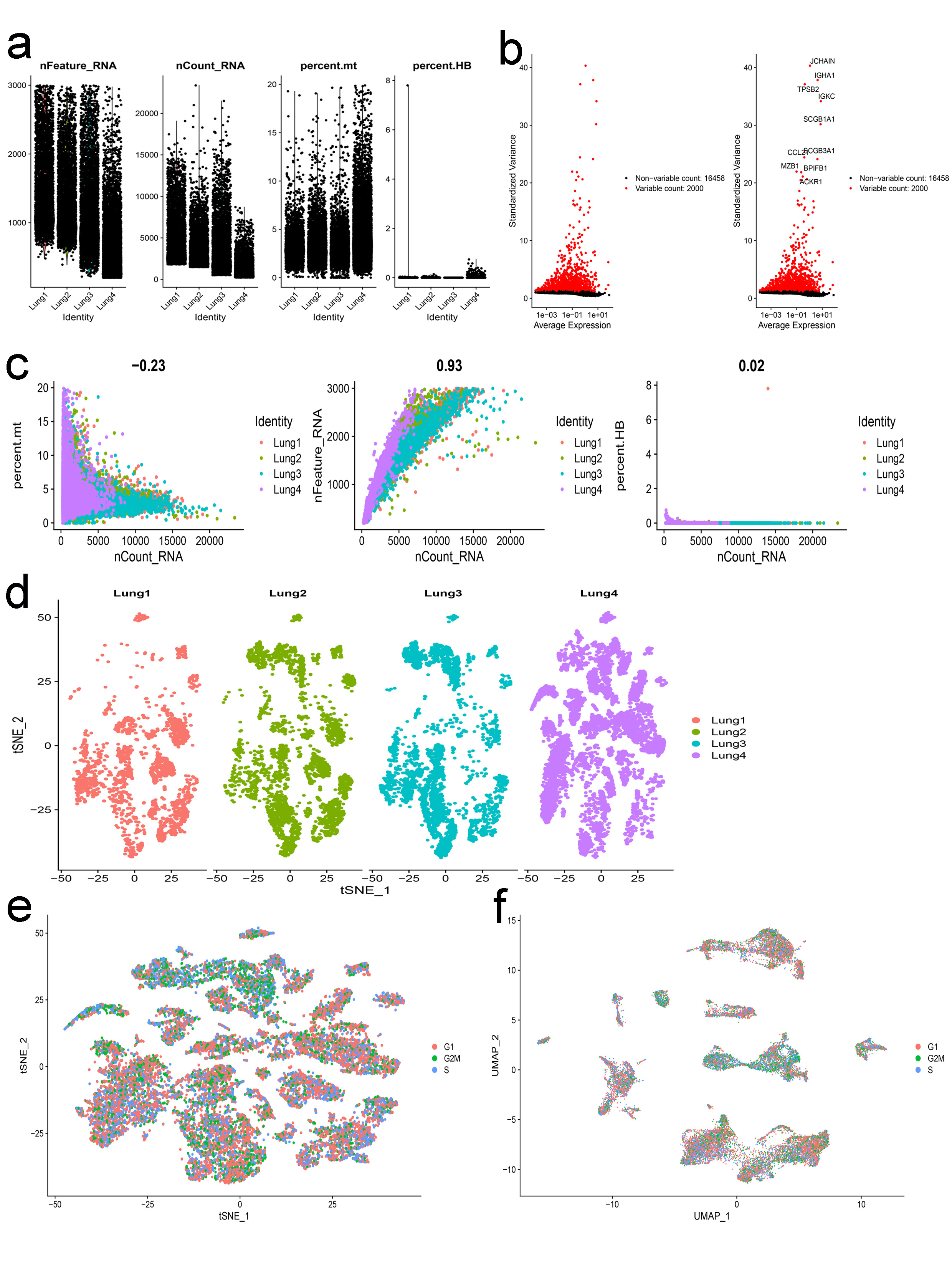

Supplement: Supplementary Figure 1 — Quality control (QC) of human lung single-cell data. (A) Scatter plot illustrating the number of genes, unique molecular identifiers (UMIs), the percentage of mitochondrial genes in each cell, and the percentage of erythrocytic genes in each cell of four lung samples. (B) 2000 genes with a high coefficient of variation between cells (Red points). (C) The relationship between the percentage of mitochondrial genes and the mRNA reads, together with the relationship between the amount of mRNA and the reads of mRNA. (D) The batch effect between four different lung samples. (E,F) tSNE and UMAP plots showing the cell cycle status of all cells. [file Image_1.JPEG]

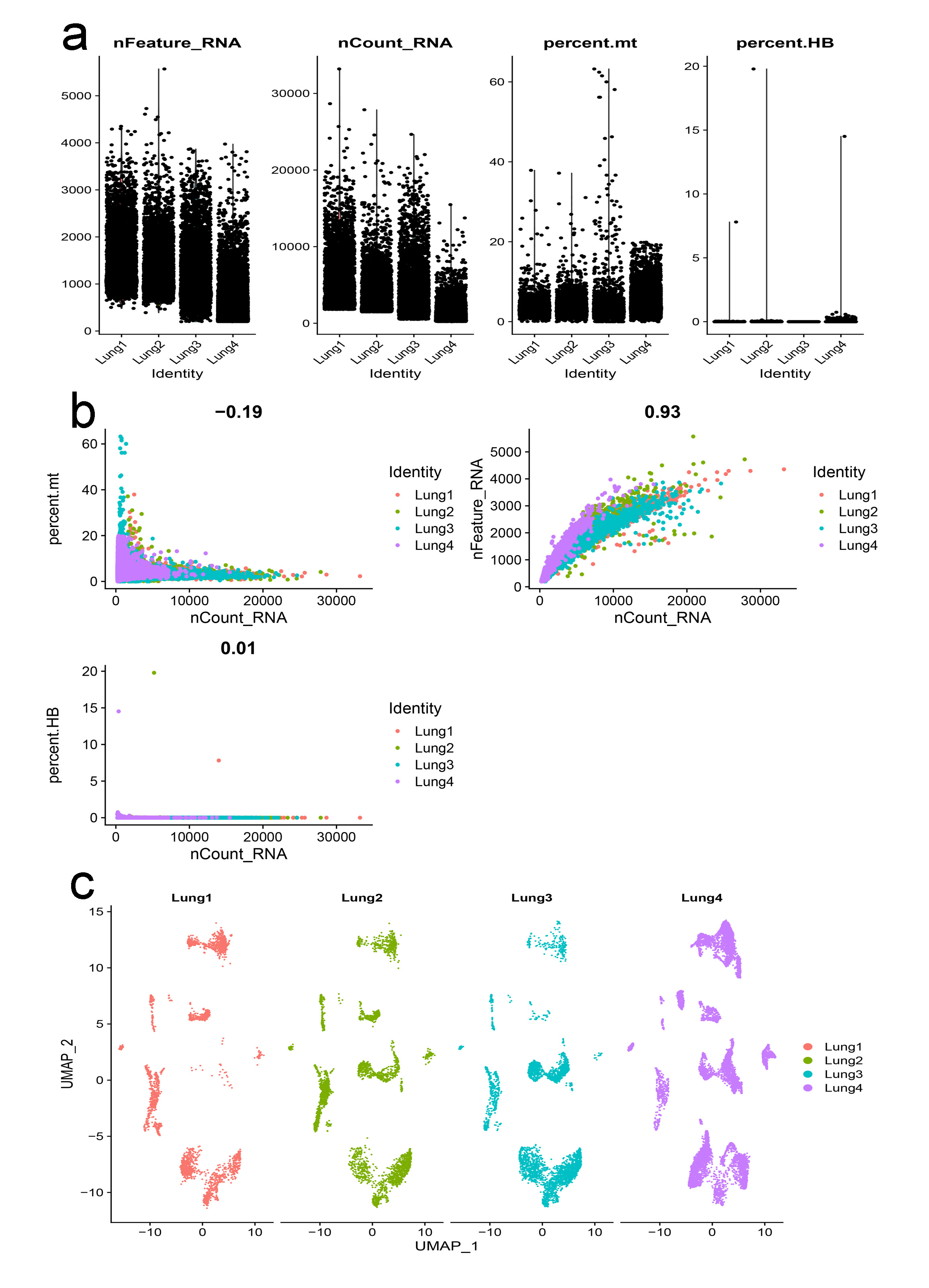

Supplement: Supplementary Figure 2 — Human lung single-cell data characteristics. (A) Before data quality control, scatter plot illustrating the number of genes, unique molecular identifiers (UMIs), the percentage of mitochondrial genes in each cell, and the percentage of erythrocytic genes in each cell of four lung samples. (B) Before data quality control, the relationship between the percentage of mitochondrial genes and the mRNA reads, together with the relationship between the amount of mRNA and the reads of mRNA. (C) After quality control, UMAP plots showing the batch effect between four different lung samples. [file Image_2.JPEG]

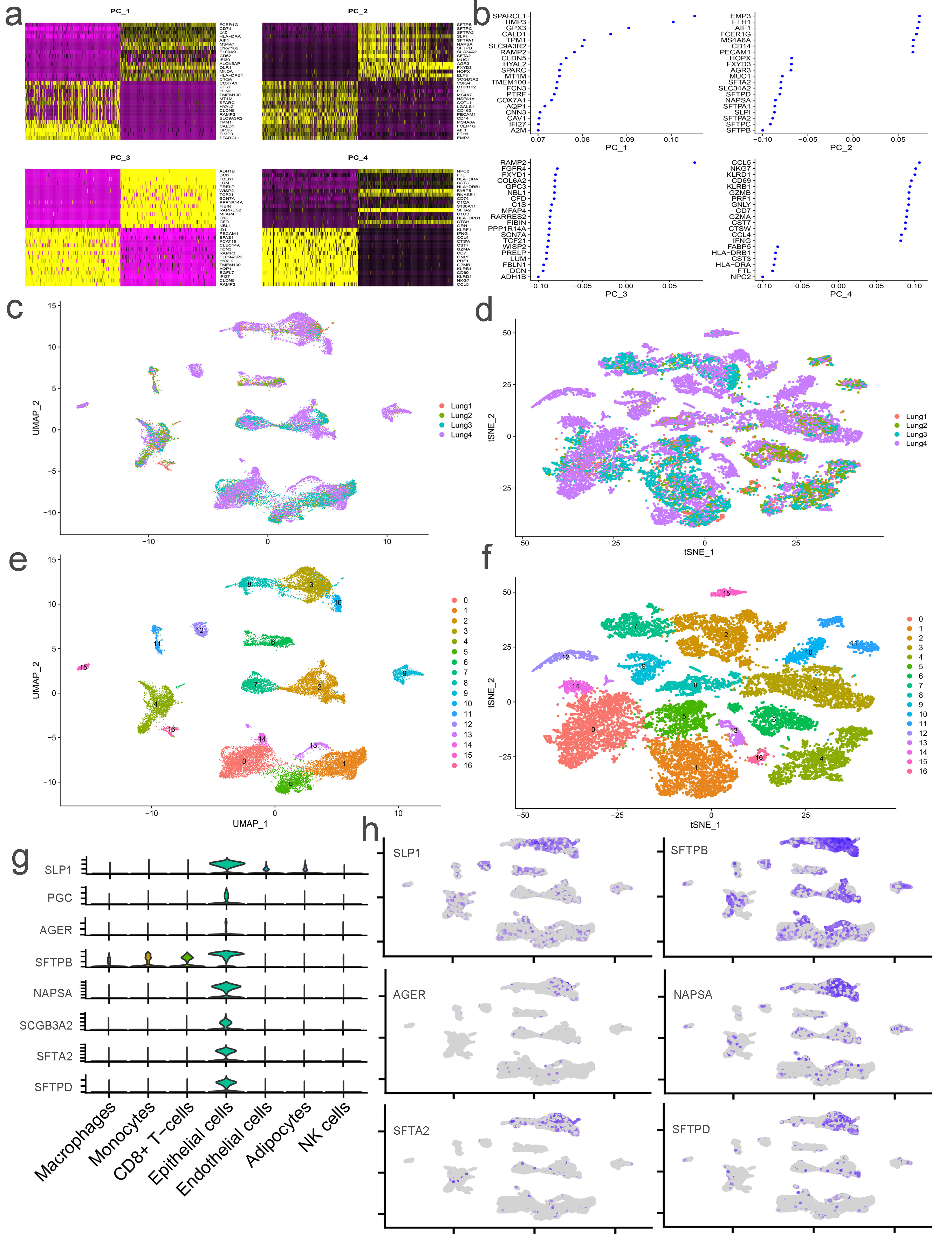

Supplement: Supplementary Figure 3 — Reduced dimension cluster analysis of scRNA-seq data of the human lung tissue. (A) Heatmap displayed the first four principal components (PCs). (B) The high variant genes in the first four PCs. (C,D) tSNE and UMAP plots showing the sample sources of cells between different cell clusters. (E,F) tSNE and UMAP plots showing lung tissue cells can be divided into 17 cell clusters. (G) Violin plots showing the expression of marker genes in epithelial cells. (H) Scatter plot of marker genes in epithelial cells. [file Image_3.JPEG]

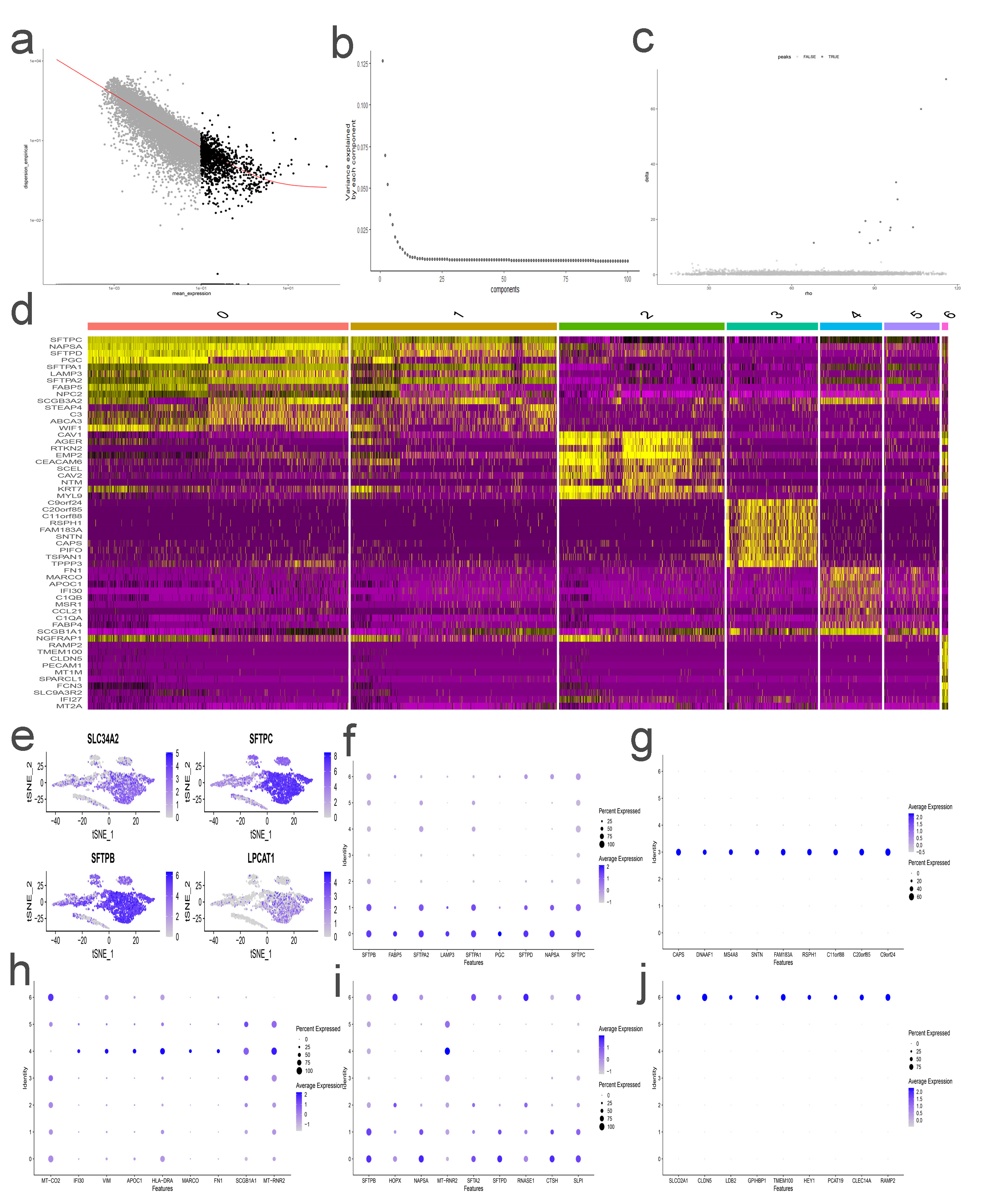

Supplement: Supplementary Figure 4 — The expression features of subpopulations of human lung epithelial cells. (A) Feature genes were selected according to the average expression level of genes (≥0.1). (B) Variation curve of difference between each principal component (PC). (C) Results of the Density Peak Cluster clustering algorithm. (D) Heatmap showing the marker genes of each cluster of epithelial cells. (E) Scatter plot of classic marker genes in epithelial cells. (F–J) Bubble plots of the first nine marker genes in each cluster of epithelial cells, (F) cluster 0&1; (G) cluster 3; (H) cluster 4; (I) cluster 5; (J) cluster 6. [file Image_4.JPEG]
